# Evidence of Cheliped Autotomy and Regeneration in the Lower Miocene Crab *Achelous monspeliensis* From Portugal

**DOI:** 10.1002/ece3.73799

**Published:** 2026-07-15

**Authors:** Carlos Neto de Carvalho, Pedro Marrecas

**Affiliations:** ^1^ Serviço de Sustentabilidade e Proteção Do Património Natural Da Câmara Municipal de Idanha‐a‐Nova; Naturtejo UNESCO Global Geopark Centro Cultural Raiano Idanha‐a‐Nova Portugal; ^2^ Sichuan Resources Group Geotourism Technology Co Chengdu City Sichuan Province China; ^3^ Universidade de Lisboa, Faculdade de Ciências Instituto D Luiz Lisboa Portugal; ^4^ Instituto de Ciências da Terra Universidade de Évora Évora Portugal; ^5^ Sociedade Portuguesa de Paleontologia Rua João Luis de Moura Lourinhã Portugal

**Keywords:** autotomy, Brachyura, defensive behavior, lower Miocene, pathologies, Portugal

## Abstract

Autotomy—the voluntary shedding of a limb as a defensive response to predation, intraspecific aggression, or infection—followed by regeneration during subsequent molting is a common behavior among decapod crustaceans. In the fossil record, however, evidence of this behavior is typically indirect and difficult to verify. In this study, we report compelling cases of autotomy, in at least one case followed by limb regeneration in *Achelous monspeliensis* from the Burdigalian deposits of Bicas–Foz da Fonte coastal section (Lower Miocene, Portugal). We based our analysis on one of the best collections of this crab species presently available for study. Among some specimens preserving pathological evidences, two crabs belonging to this homochelate species exhibit partial regeneration of cheliped characterized by marked dimensional asymmetry of the podomeres. The aborted regenerative sequence indicates that they died before completing the number of molts required to fully restore the limb to its original size.

## Graphical abstract

Crab autotomy with evidence of regeneration is described for the first time in the fossil record. Autotomy is a defensive behavior that affects more males than females of *Achelous monspeliensis* and may be related to mating season.

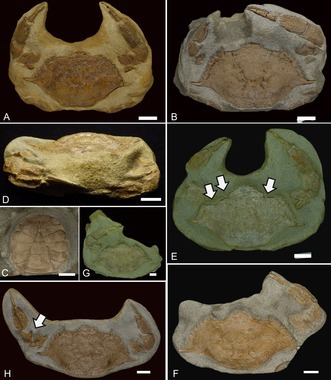

## 1 Introduction

Brachyurans, the so‐called “true crabs,” can be considered among the most evolutionarily successful crustaceans due to their exceptional species diversity, wide geographic distribution, and ability to exploit an extraordinary range of ecological niches (Ng et al. 2008; Tsang et al. 2014; Lee 2016). Their characteristic body plan—a compact carapace combined with robust, heavily calcified multifunctional chelae—has facilitated both defense and specialization in feeding strategies, contributing to their adaptive radiation (Wolfe et al. 2021; Koneru and Caro 2025). Moreover, brachyurans occupy environments from deep‐sea vents to terrestrial forests, demonstrating notable physiological and behavioral flexibility (Lee 2016; Turner 2016; Wolfe et al. 2024). In the fossil record, however, brachyurans are usually known only from chelae or isolated dactyli, as these heavily mineralized elements generally persist long after carcasses disarticulate and the lightly calcified carapace dissolves (Krause et al. 2011; Jagt et al. 2016; Klompmaker et al. 2017). The dense mineralization of chelae allows them to withstand post‐mortem transport, scavenging, and early diagenetic dissolution far better than the remaining, more lightly calcified carapace. Therefore, the scarcity of complete specimens reflects both anatomical properties and the depositional environment in which the crab remains accumulate.

Nearly complete crab carcasses are exceptional occurrences as only under unusually favorable taphonomic conditions that minimize decay, disarticulation, and dissolution, can be found (Allison 1986; Matos et al. 2021). Such preservation typically requires rapid burial in fine‐grained sediments within low‐energy, oxygen‐poor settings, where scavenging and microbial activity are greatly reduced (e.g., Kidwell and Flessa 1996). Anoxic or dysoxic bottom waters, coupled with limited bioturbation, help maintain the integrity of the exoskeleton long enough for early diagenetic mineralization to stabilize the lightly calcified carapace. In some cases, natural shelters such as burrows also protect the carcass at the moment of death. Together, these factors create the rare circumstances under which crabs can enter the fossil record as nearly complete specimens rather than as isolated, heavily mineralized elements.

The study of exceptionally preserved crab body fossils, together with their trace fossils and other evidences of behavior, provide a uniquely powerful window into the evolutionary paleobiology of Brachyura (Carmona et al. 2004; Gibert et al. 2013). Complete or nearly complete carcasses reveal fine morphological details—such as carapace ornamentation, limb proportions, sensory structures, and soft‐tissue impressions (e.g., Luque et al. 2021)—that are otherwise lost in the fragmentary fossil record, allowing more accurate reconstructions of phylogeny, functional anatomy, and ecological specialization. Trace fossils, including burrows, trackways, and feeding structures, as well as other forms of behavior that may be interpreted from the body fossil record, especially when the study is focused on large samples and/or exceptionally preserved examples, complement this information by documenting behaviors that individual body fossils alone cannot capture, such as locomotion, sediment interaction, predation strategies and other inter‐ and intraspecific interactions, and habitat preferences (e.g., Turner 2004; Carmona et al. 2004; Benton 2010; De Baets and Littlewood 2015; Klompmaker and Boxshall 2015; Klompmaker et al. 2019; Lindgren et al. 2025). When integrated, these lines of evidence illuminate how crabs diversified, adapted to new environments and ecological constraints, and co‐evolved increasingly complex behaviors through time. They also help calibrate evolutionary timelines and identify key ecological transitions (Neto de Carvalho et al. 2010; Luque et al. 2019, 2021, 2024; Baucon et al. 2025)—ultimately offering a far more complete picture of crab evolution than either record could provide independently.

Autotomy, the voluntary shedding of a limb during predation, intraspecific aggression, or following injury, is a behavior widespread among modern decapods, which commonly regenerate lost appendages (Wood and Wood 1932). Fossil evidence, however, has been only circumstantial (Feldmann 2003; Luque et al. 2021). Here we provide the first compelling documentation of autotomy followed by regeneration in fossil crabs. Burdigalian specimens of *Achelous monspeliensis* are highlighted from a collection of exceptionally preserved crabs of this species showing some abnormalities of physiological development (pathological individuals), by exhibiting predation marks and loss of one cheliped that may have resulted in death. However, few specimens show partial cheliped regeneration with clear dimensional asymmetry, indicating that the individuals died before completing the number of molts necessary to fully restore the limb, consistent with regenerative patterns observed in extant *Achelous* and *Portunus* species *sensu lato*.

### 1.1 Previous Studies on Exceptionally Preserved Decapod Fossil Record From Portugal

The earliest evidence of crabs in Portugal can be attributed to Middle Jurassic crab‐like locomotion traces from the S. Bento “Jurassic Beach” (upper Bajocian; Neto de Carvalho et al. 2016). Brachyuran crab body fossils first appear in the mid‐Oxfordian Cabaços Formation (Wehner 1988; Müller et al. 2000). Although marine Cretaceous units have high potential for crustacean preservation, remains of crabs have not yet been taxonomically revised, despite exceptionally preserved mass burials of the mecochirid lobster *Atherfieldastacus rapax* being known in Barremian units (Neto de Carvalho et al. 2007; Neto de Carvalho 2016).

Neogene crabs are better documented, beginning with Fontannes (1884) and later studies by Veiga Ferreira (1954, 1958, 1965), Galopim de Carvalho (1959), Zbyszewski and Veiga Ferreira (1962), and Kotchetoff et al. (1975), who recorded a range of taxa from Lower and Middle Miocene deposits from the Baixo Tejo Basin. Recent work from Penedo Norte section, an important crustacean site, added the species *Calappilia erwinhartei* (Wallaard et al. 2020), which may fall in synonymy with *Calappilia matzkei* (Àlex Ossó, pers. commun.). More recently, Neto de Carvalho and Marrecas (2022) preliminarily described the first clear evidence of autotomy followed by regeneration in the still described as *Portunus monspeliensis* from the Burdigalian of Bicas‐Foz da Fonte (Lower Miocene, Portugal), based on a very limited sample size.

Our present study extends the previous work and focuses on the occurrence of abundant crabs in nodules from the Bicas‐Foz da Fonte coastal section, which is in stratigraphic continuation of the Penedo Norte fossil site. We have looked for possible explanations to explain the unusual disproportion between male and female crabs in the thanatocoenoses, including looking for pathological evidences in individuals during their life span to explain possible causes of death.

### 1.2 Stratigraphic Context

**FIGURE 1:**
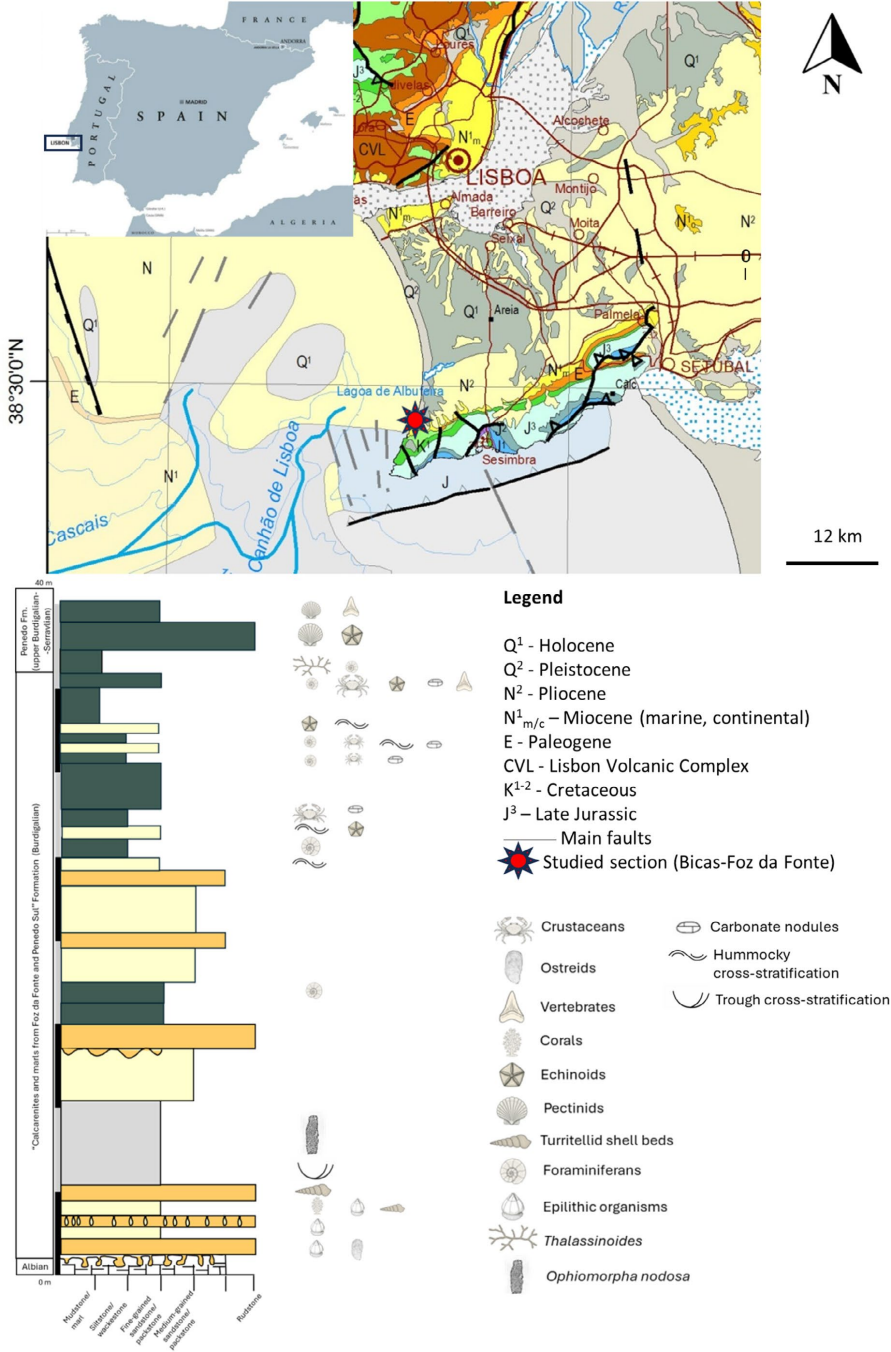
Simplified geological map of the Lower Tejo River region, with the distribution of the main Miocene deposits (extract adapted from the geological map of Portugal at 1:1,000,000 scale edited in 2010 by Laboratório Nacional de Energia e Geologia, I.P); symbol indicates the approximate location of the studied locality. The simplified stratigraphic log provides the distribution of the main beds with crab nodules.

The coastal cliffs between Penedo and Foz da Fonte beaches constitute a reference section for the Miocene of the Baixo Tejo Basin (Pais et al. 2012), recording a shallow‐marine carbonate to mixed carbonate–siliciclastic depositional system that is regionally condensed and locally interrupted by hiatuses. These deposits belong to the southern sector of the Mio‐Pliocene Baixo Tejo Basin (Setúbal Peninsula) and form part of the northern limb of the fold–fault system associated with the Betic‐oriented Arrábida mountain chain, which develops further to the southeast (Figure 1).

**FIGURE 2:**
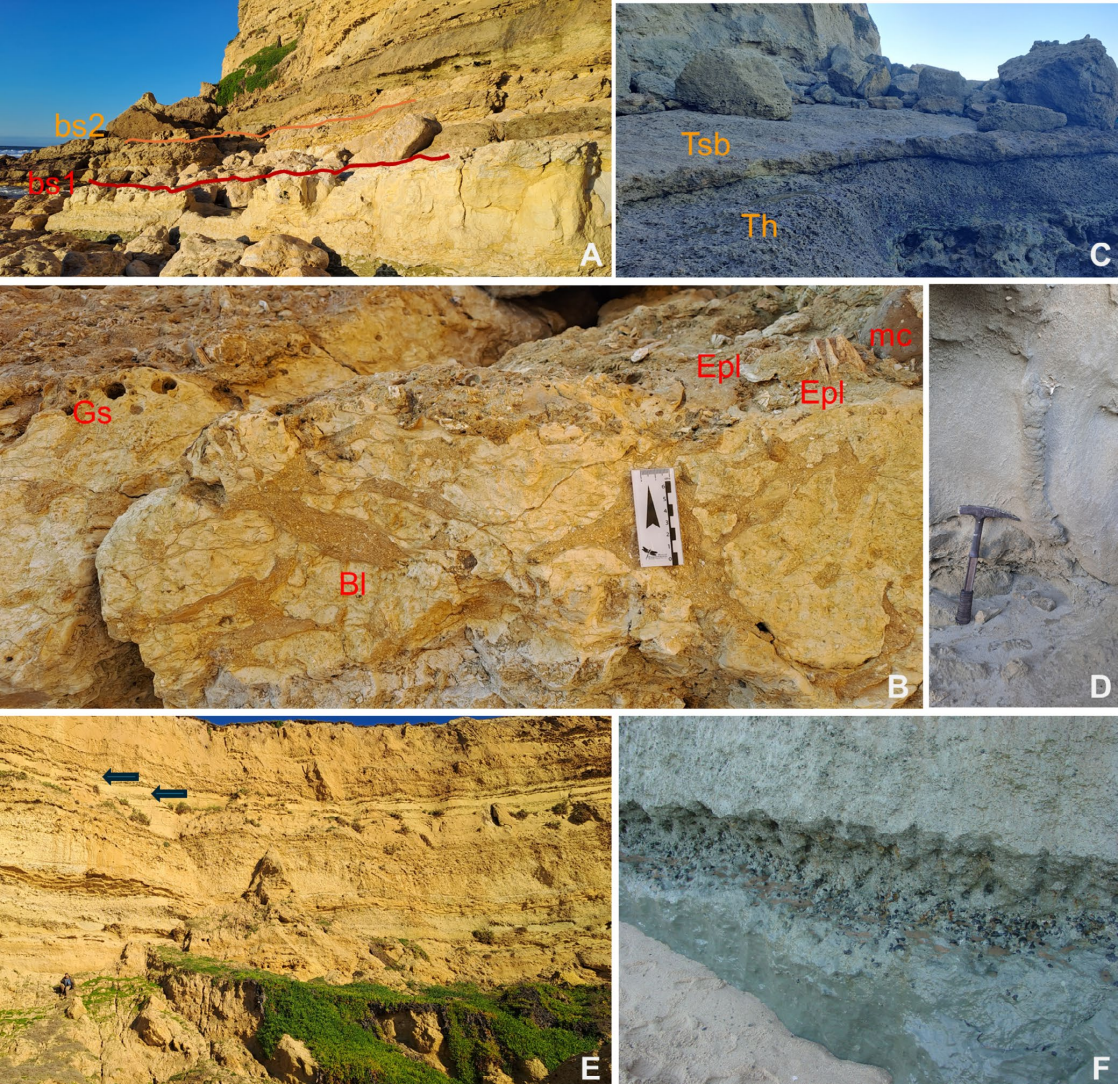
Stratigraphic features of the Bica‐Foz da Fonte and Penedo Norte sections. (A) Bioerosional surfaces marking an angular unconformity separating the Albian limestones from the “Calcarenites and marls from Foz da Fonte and Penedo Sul” Fm. (bs1), and an intra‐early Burdigalian hiatus with the formation of a rocky shore (bs2). (B) Detail of bs2 showing *Gastrochaenolites* (Gs), epilithic organisms including barnacles (Epl) and clasts of Cretaceous magmatic rocks (mr); the bed whose bedding plane was bioeroded shows evidence for the development of a hardground with the presence of *Balanoglossites triadicus*. (C) Packstones densely bioturbated with *Thalassinoides* (Th) and *Turritela* shell beds (Tsb). (D) *Ophiomorpha nodosa* in coarse‐grained sandstones. (E) The southernmost Foz da Fonte section with the location of the beds with crab carbonate nodules in the middle interval of the “Calcarenites and marls from Foz da Fonte and Penedo Sul” Fm. (F) Penedo Norte section: Phosphate nodule bed at the top of the “Calcarenites and marls from Foz da Fonte and Penedo Sul” Fm.; crab nodules were retrieved in situ in the bioturbated marlstone just below.

The studied interval corresponds to the “Calcarenites and marls from Foz da Fonte and Penedo Sul” unit of Manuppella et al. (1999), which reaches a maximum thickness of ca. 35 m. These deposits rest unconformably on Albian (Lower Cretaceous) strata along an angular unconformity marked by intense bioerosion (Santos et al. 2010). The unconformity records a major stratigraphic break preceding Burdigalian marine transgression (Figure 2A).

The lower interval (0–12 m) is characterized by high‐energy, carbonate and siliciclastic shoreface deposits. It includes evidence of at least one more bioerosional surface (bs2) showing encrusting epibionts (Figure 2B), overlain by coarse, storm‐reworked biocalcarenites rich in rhodoliths and abundant ghost‐shrimp burrows (*Ophiomorpha nodosa*; Figure 2C). *Turritella* shell beds, intense thalassinidean shrimp bioturbation (*Thalassinoides*; Figure 2D) identifying firmgrounds, intercalated with cross‐lamination and storm‐generated packstone textures, indicate episodic high‐energy reworking on a shallow offshore transition.

The middle interval (12–20 m) consists predominantly of siltstones and marly facies, recording a transition to quieter, more offshore depositional conditions. This interval reflects a reduction in hydrodynamic energy and increased siliciclastic input. Most of the carbonate nodules bearing crab fauna originate from these beds (Figure 2E), indicating rapid encapsulation from Ca‐rich pore waters, protective fine‐grained matrix and sustained burial below disturbance depth (Kidwell et al. 1986; Behrensmeyer et al. 2000).

The upper interval (20–35 m) comprises mixed carbonate–siliciclastic facies with abundant and diverse marine macrofauna. These heterolithic deposits reflect an offshore transition. Thin marly interbeds and localized siliciclastic layers record short‐lived shifts to more distal, muddier or lower‐energy offshore conditions. In this facies, near‐complete sirenian and articulated cetacean skeletons were found. Calcareous nannofossil assemblages from this interval are consistent with fully marine neritic conditions (Figueiredo et al. 2023). In the upper part of this interval, crab nodules were found, and phosphate nodules and fossils and glauconite‐rich beds are common, as in the overlying Penedo Formation (Figure 2F).

The exposed strata in the Bicas‐Foz da Fonte section have been consistently assigned to the Burdigalian based on integrated biostratigraphic, magnetostratigraphic, and isotopic data (Zbyszewski 1967; Antunes et al. 1998, 1999; Manuppella et al. 1999; Cachão and da Silva 2000; Silva et al. 2000). In effect, strontium isotope ratios (^87^Sr/^86^Sr) obtained from these deposits indicate ages between ca. 20 and 17.6 Ma, confirming an early‐to‐middle Burdigalian age for the “Calcarenites and marls from Foz da Fonte and Penedo Sul” Formation (Antunes et al. 1995). At the top of the succession, extending into the base of the Penedo section, the presence of common *Helicosphaera ampliaperta* associated with rare *H. magnifica* constrains this interval to calcareous nannofossil biozone NN3 (Martini 1971), approximately equivalent to the middle Burdigalian (Figueiredo et al. 2023).

## 2 Materials and Methods

Dozens of nodules containing exceptionally well‐preserved crabs were discovered by one of the authors (P.M.) in biocalcarenite and marl levels between Bicas and Foz da Fonte beaches (Figure 1). The specimens analyzed here were recovered from these carbonate nodules, which become detached from the sea cliffs by continuous differential erosion along the coast. These nodules occur in the intertidal zone and are rapidly destroyed by wave action. Although only a few of the collected specimens were recovered in strict stratigraphic context, all come from the medium and upper parts of the “Foz da Fonte and Penedo Sul Calcarenites and Marls” unit (Manuppella et al. 1999) and are representatives of different populations. GPS locality information is available from the authors upon request.

After collection, the nodules required desalination to ensure long‐term preservation. This was achieved by immersing them in fresh water—changed frequently—over several weeks. Preparation was carried out by P.M. using pneumatic micro‐hammers (JQS‐108A with a sharpened tungsten tip at a narrower angle, and Borntun BD‐0086 fitted with an extra‐long tungsten needle manufactured by DNSONS TOOLS). As morphological features of the crabs were exposed, a solution of Paraloid B‐72 in acetone was applied progressively. At the end of preparation, all specimens were consolidated with a 10% Paraloid B‐72 solution in acetone.

**FIGURE 3:**
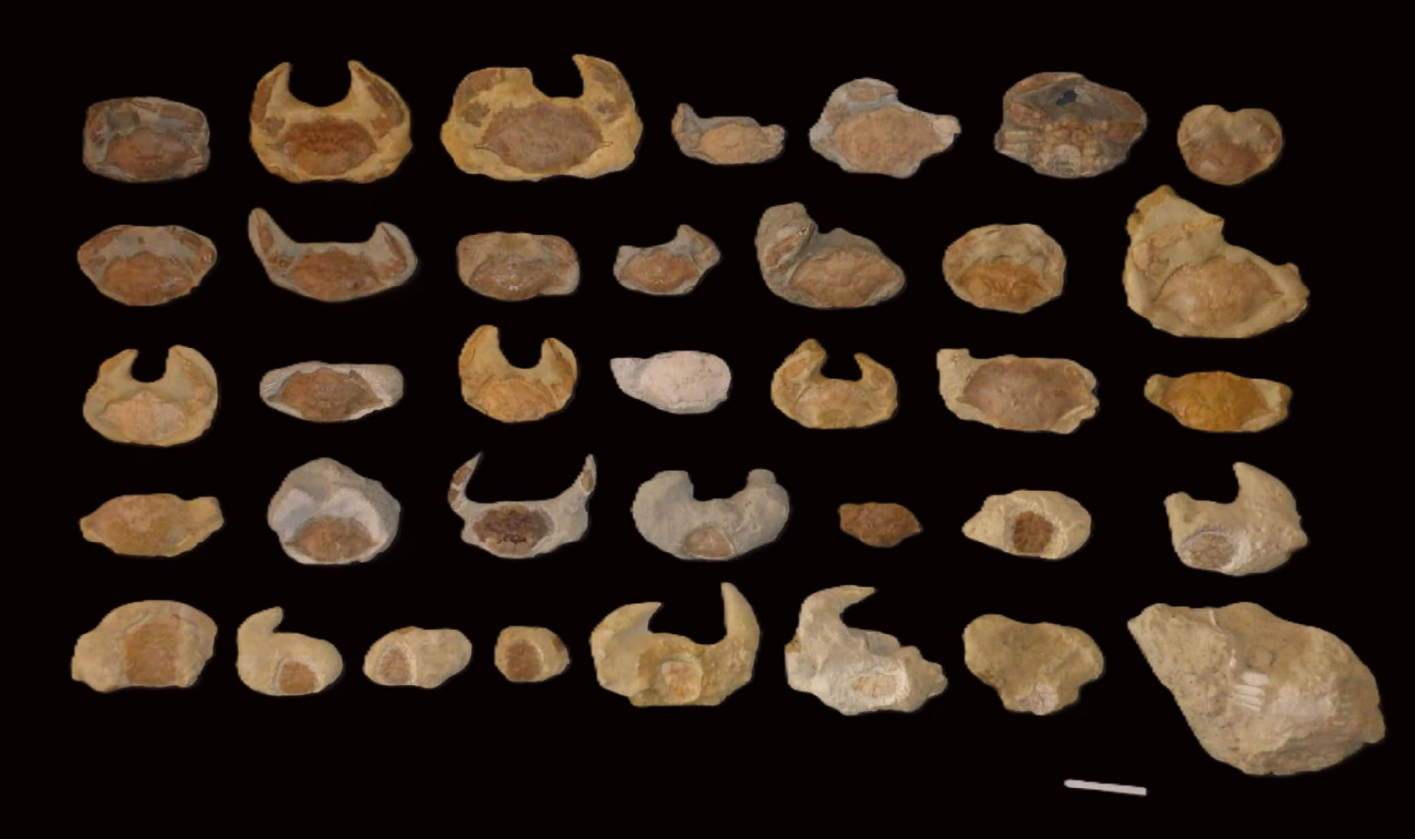
General view of the collection of exceptionally preserved *A. monspeliensis* used in the present study. From top to bottom and from left to right the specimens were numbered CPM–BFF001D to ‐BFF036D. Scale is 50 mm.

**FIGURE 4:**
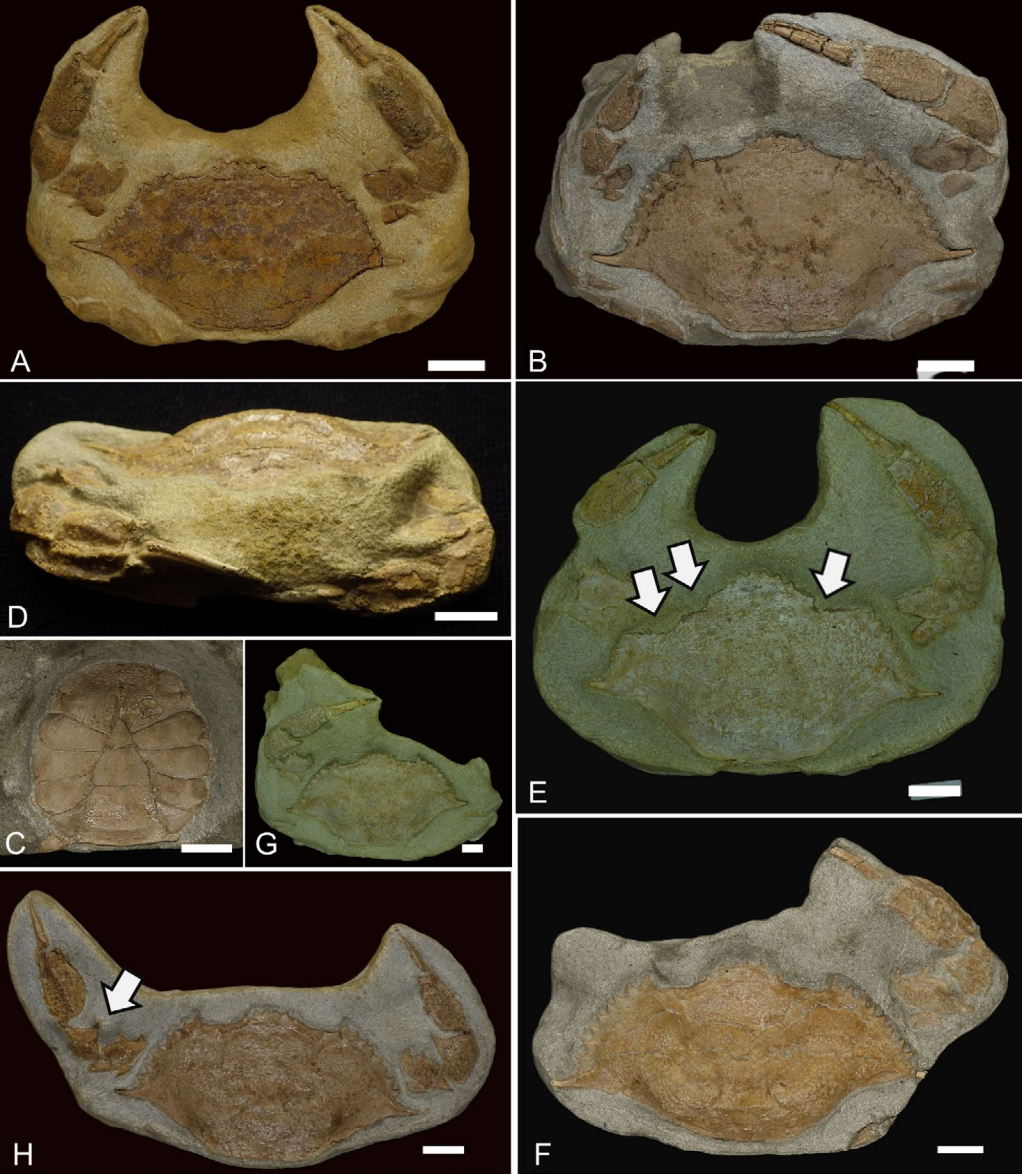
General morphological features of *A. monspeliensis* from the Burdigalian of Bicas‐Foz da Fonte sector in Portugal, and the exceptional variations: (A) Characteristic adult specimen of *A. monspeliensis* Weber (CPM‐BFF002D: Dorsal view); (B) Autotomized specimen (dorsal view), as interpreted by the noticeably smaller dimensions of the podomeres from the left cheliped in comparison with the right cheliped (CPM‐BFF001D); (C) The same specimen in ventral view to highlight the morphology of the somites in a male individual. (D) Frontal view of the specimen CPM‐BFF010D showing the chelipeds displacement in different planes which result in an apparent morphological asymmetry; (E) Pathologies in both sides of the anterolateral margin of the carapace that may have resulted from predation, or from aggressive behavior between male crabs (CPM‐BFF015D); (F, G) One‐limb crabs possibly related to autotomy just before death (CPM‐BFF011D and CPM‐BFF014D, respectively); (H) CPM‐BFF009D with malformation of the carpus (arrow) that may have been responsible for the “nearly stretched” arrangement of the left cheliped. Graphic scale is 10 mm.

For the present study, 36 exceptionally‐preserved specimens identified as *Achelous monspeliensis* and carefully prepared—numbered CPM–BFF001D to BFF036D—were used, with a subset of 24 more complete specimens selected for the taxonomic and morphometric measurements (Table 1; Figure 3). The primary specimen discussed herein is cataloged as CPM–BFF001D (Figure 4A). The measurements were obtained by using a manual caliper. The collection is private but open to research upon request to P.M.

**TABLE 1:** Specimens and measurements used in the study of *A. monspeliensis* from the Burdigalian sea cliffs of Bicas–Foz da Fonte.

Specimen no. ♂ | ♀	Carapace width (mm)	Carapace length (mm)	Frontal width (mm)	Frontorbital width (mm)	Posterior width (mm)	Merus length (mm), left/right		Carpus length (mm), left/right		Propodus length (mm), left/right	
CPM‐BFF001D ♂	51	32	10.5	30	20	7	12.5	8	11.5	9	15
CPM‐BFF002D ♂	60	37	12.5	32	23	16	16	12	12	16	17
CPM‐BFF003D ♂	74	43.5	14	39	27	20	20	15	14	22	20
CPM‐BFF004D ♀	43	25.5	8.5	24.5	18	8	10	7	—	10	—
CPM‐BFF005D ♂	67.5	43	13	37	25	—	—	—	—	—	—
CPM‐BFF006D ♀	—	—	—	—	—	—	—	—	—	—	—
CPM‐BFF007D ♂	42.5	26	8.5	25	19	10.5	9.5	7	8	11	11.5
CPM‐BFF008D ♀	53	32.5	11	32.5	21	13	12	11	11	14	14.5
CPM‐BFF009D ♂	54.5	31	11	31.5	21.5	11	11	7	10	14	13
CPM‐BFF010D ♀	52	29	10	30	23	17	12	—	12	13.5	15
CPM‐BFF011D ♂	47	25	10	26	16	—	11	—	8	—	13
CPM‐BFF012D ♂	64	39	13	36	26	15.5	—	13	—	16	—
CPM‐BFF013D ♂	51	32	10	29	21	12	12	11	—	14	—
CPM‐BFF014D ♂	71	42	14	38	29	19	—	15	—	22	—
CPM‐BFF015D ♂	50	32	10.5	—	20	—	14.5	—	12	—	16
CPM‐BFF016D ♂	58	33.5	12	33	25	—	—	—	—	—	—
CPM‐BFF017D ♂	50	—	10	28	—	11	—	10	—	13	—
CPM‐BFF018D ♂	62	—	13	35	—	—	—	—	—	—	—
CPM‐BFF019D ♂	50	—	11	29	—	13	12	11	10	14	13
CPM‐BFF020D ♂	76	44	14	38	25	—	—	—	—	—	—
CPM‐BFF021D ♂	63	38	13.5	35	26	—	—	—	—	—	—
CPM‐BFF022D ♂	66	—	—	—	24	—	—	—	—	—	—
CPM‐BFF023D ♀	45	—	—	—	21	—	—	—	—	—	—
CPM‐BFF024D ♂	59	33.5	10.5	31	19.5	15	16	11.5	11	15	16
CPM‐BFF025D ♂	—	—	—	—	—	—	—	—	—	—	—
CPM‐BFF026D ♂	46	27.5	9	26.5	16	—	—	—	—	—	—
CPM‐BFF027D ♂	—	—	—	—	—	—	—	—	—	—	—
CPM‐BFF028D ♂	—	—	—	—	—	—	—	—	—	—	—
CPM‐BFF029D ♂	—	—	—	—	—	—	—	—	—	—	—
CPM‐BFF030D ♂	—	—	—	—	—	—	—	—	—	—	—
CPM‐BFF031D ♂	—	—	—	—	—	—	—	—	—	—	—
CPM‐BFF032D ♀	—	—	—	—	—	—	—	—	—	—	—
CPM‐BFF033D ♀	—	—	—	—	—	—	—	—	—	—	—
CPM‐BFF034D ♀	—	—	—	—	—	—	—	—	—	—	—
CPM‐BFF035D ♀	—	—	—	—	—	—	—	—	—	—	—
CPM‐BFF036D ♀	—	—	—	—	—	—	—	—	—	—	—

Most of the specimens recovered from the nodules belong to a single species of the portunid swimming crab *A. monspeliensis* (see Systematic Paleontology at the end of this paper and Figure 4). Only three specimens refer to a panopeid crab and one very incomplete specimen to a raninid crab and will not be considered in the present study. Besides, we also identified two dactyli of a paguroid hermit crab, probably the typical “*Petrochirus priscus*,” dactyli of a calappid box crab, and one dactyl and palm of a ghost shrimp callianassid (Àlex Ossó, person. commun.). Their preservation is exceptional, despite the rapid erosion affecting the nodules, and typically includes both chelipeds (one or the two chelipeds may be already partially ablated by recent erosion) with their full spiny ornamentation as well as the proximal portions of most pereiopods. The exocuticle is generally absent, having dissolved during early diagenesis and contributed to the formation of the carbonate film that encloses the fossils. However, in the upper beds of Bicas‐Foz da Fonte section and the basal levels of the Penedo succession, in the northward continuation of the Bicas‐Foz da Fonte section and the uppermost part of the Foz da Fonte and Penedo Sul Calcarenites and Marls” succession, two specimens of *A. monspeliensis* retain remnants of the exocuticle on the dorsal carapace—dark brown in color and showing coarse granular ornamentation (e.g., CPM‐BFF024D in the studied sample). A third example comes from the yellow biocalcarenite beds located in the upper part of the Bicas‐Foz da Fonte section, where the exocuticle was replaced by carbonate (CPM‐BFF036D). Together with the lack of pereiopod disarticulation, this indicates that the *A. monspeliensis* individuals studied here died and were preserved in situ, most likely due to environmental causes (sensu Gašparič et al. 2019).

### 2.1 Discussion: Evidence for Cheliped Autotomy With Regeneration

Among the sample of 36 specimens we identified five individuals that may show pathological conditions: CPM‐BFF015D presents injuries that affected the anterolateral margin in both sides of the orbital region (Figure 4E), whereas CPM‐BFF011D and CPM‐BFF014D lack one cheliped in each (Figure 4F,G). We don't know if these pathologies were produced during life, are related to the cause of death, or resulted post‐mortem. On the other hand, CPM‐BFF001D and CPM‐BFF009D show dimensional asymmetry of podomeres (Figure 4B,H), with the latter resulting in a left carpus smaller and deformed. These cases are interpreted here as non‐lethal autotomized individuals where regeneration was not fully achieved.

Loss and regeneration of the walking legs, especially the first appendages, are widespread within arthropods and well documented in extant decapod crustaceans (Wood and Wood 1932; Maruzzo et al. 2005). Non‐lethal predation marks have been recognized in the fossil record of mollusks, crinoids, ophiuroids, ammonoids, nautiloids, and trilobites (Klompmaker et al. 2019). These authors also described regenerated crinoid arms interpreted as evidence of failed predation attempts or autotomy behavior triggered by environmental stress. However, evidence for autotomy in decapod crustaceans—particularly in brachyuran crabs—is mostly circumstantial (Förster 1969; Feldmann 2003). The exceptionally preserved crustaceans from the Terrain à Chailles Formation provide strong evidence for autotomy in Jurassic decapods, particularly in *Glyphea regleyana*, and more tentatively inferred for *Eryma ventrosa* and *Glyphea muensteri* (Charbonnier et al. 2012). This evidence is based on the recurrent occurrence of isolated first pereiopods preserved without associated carapaces, often as complete and symmetrical elements, indicating selective limb loss rather than random post‐mortem disarticulation. The inferred breakage point corresponds to the basis–ischium articulation, a known autotomy plane in extant glypheid lobsters. Comparisons with the living *Neoglyphea inopinata* , in which loss of the first pereiopods is frequent and interpreted as a stress‐ or predator‐induced defensive reflex, strongly support a similar biological mechanism in the fossil taxa. Together, these observations represent one of the clearest demonstrations of autotomy in the crustacean fossil record and extend the evolutionary origin of this antipredatory behavior back to at least the Late Jurassic (Charbonnier et al. 2012). Luque et al. (2021) recently described a ~100 Ma Cretaceous crab from Myanmar preserved in amber with one locomotory appendage detached; the authors suggest that the limb may have been autotomized at the moment the animal became trapped in resin.

Autotomy may be understood as a defensive interaction between the crab and its environment at any life stage, beginning in the juvenile phase. This behavior may lead to the fracture of a pereiopod, potentially followed by infection, or may occur in response to imminent predatory attacks or aggressive intraspecific interactions. Known fossil predators of decapods across time include fishes, plesiosaurs, ammonites, octopods, and gastropods (Klompmaker et al. 2019).

Although the absence of one or more locomotory appendages in fossil crabs may result from multiple taphonomic or preservational processes, as in the cases of the CPM‐BFF011D (without left cheliped; Figure 4F) and CPM‐BFF014D (without right cheliped; Figure 4G)—and is therefore insufficient evidence for autotomy—the occurrence of homochelate individuals with one abnormally small pereiopod or smaller podomeres may reflect partial regeneration interrupted by the death of the animal. Feldmann (2003) emphasized that abnormally small limbs in fossil crabs are not diagnostic of autotomy; unequivocal regeneration would require evidence of a malformed limb that developed abnormally during growth. Nevertheless, studies of several extant crab species, including multiple species of *Portunus* (e.g., Hopkins 2001; Paterson et al. 2007; He et al. 2016; Fu et al. 2017; Liu et al. 2018; Triay‐Portella et al. 2018), show that limbs lost via reflexive autotomy may be completely regenerated within a single molting cycle. In *P. trituberculatus*, He et al. (2016) documented three regeneration stages following autotomy of a cheliped: (i) immediately after autotomy, without visible limb bud formation; (ii) beginning of the molt, with formation of the limb bud; and (iii) completion of the molt, producing a slightly smaller but morphologically normal regenerated appendage.

Autotomy typically occurs at a predetermined breakage plane at the base of each appendage, between the coxa and the proximal ischium, regardless of which distal segment is damaged. This joint is non‐functional (Wood and Wood 1932; Bliss 1960). Complete limb regeneration from this preferential breakage plane has been documented in many crab species (Bliss 1960; Vernet and Charmantier‐Daures 1994). Until the subsequent molt, an external cuticular sheath seals the joint and encloses the developing limb bud (Smith 1995). Limb growth proceeds through alternating slow and rapid phases involving mitotic proliferation, protein and water accumulation, and joint differentiation. During the molt following autotomy, the regenerated limb emerges from the cuticular sheath. Full limb size is usually achieved within two or three molts, though in some species regeneration can be completed in a single molt (Ameer Hamsa 1982; He et al. 2016). The rate and success of regeneration depend on molt frequency and the degree of growth achieved in each instar; these factors decrease with age and may cease entirely once the animal reaches terminal anecdysis (Juanes and Smith 1995). If limb loss occurs outside the predetermined breakage plane, regeneration is slower, less efficient, and may remain incomplete.

In the adult specimen of *A. monspeliensis* CPM‐BFF001D, the left cheliped shows no signs of abnormal growth or diagenetical deformation; only the relative dimensions of its segments are significantly smaller than those of the right cheliped. Specifically, the merus, carpus, and palm left/right ratios are 0.56:1, 0.70:1, 0.9:1, respectively. This proportional reduction supports interpretation of the left cheliped as a normally regenerating appendage following reflexive autotomy at the preferred breakage plane near the base of the ischium. Typically, injuries limited to the movable fingers or claws do not trigger full‐limb autotomy (Juanes and Smith 1995). CPM‐BFF009D, where the left carpus is malformed and is smaller than the right one (Figure 4H), may be one of such cases. In the CPM‐BFF001D, however, the reduced size of all segments suggests that the animal died shortly after autotomy, before complete regeneration could occur in one or more subsequent molts.

### 2.2 Possible Behavioral and Ecological Causes for Autotomy in *A. monspelliensis*

High frequencies of limb autotomy—especially of chelipeds—have been observed in juvenile *P. trituberculatus* (He et al. 2016), but the behavior occurs throughout ontogeny in this and other portunid extant species (Smith 1995; Juanes and Smith 1995; Triay‐Portella et al. 2018), and in both sexes. In these cases, autotomy has been shown to be an effective escape response to intraspecific aggression, including cannibalism.

Complete limb loss results in reduced body size (measured as carapace width) in *P. pelagicus* after molting (Paterson et al. 2007). Autotomy decreases growth rate during molt, alters the timing of ecdysis, and reduces feeding efficiency. It may impair prey handling, reproductive success, and increase vulnerability to both intra‐ and interspecific attacks (Juanes and Smith 1995). Even so, autotomized individuals of *P. pelagicus* are known to adjust their feeding strategies, becoming more herbivorous, for example (Juanes and Smith 1995). Given the variability in maximum carapace width for the autotomized specimens described here (between 47 mm for CPM‐BFF011D and 71 mm for CPM‐BFF014D) within the small sample examined—and considering that the specimens derive from different stratigraphic layers representing distinct populations—it is not possible to determine whether the autotomized *A. monspeliensis* individuals with clear evidences of regeneration CPM‐BFF001D and CPM‐BFF009D are significantly smaller than the others, despite their width/length ratio being clearly variable (1.59 and 1.76, respectively) to the mean value obtained from the sample (1.68).

Besides the pathological conditions, and despite the small number of specimens, we found a large disproportion between male and female death rates among *A. monspeliensis* in the thanatocoenoses from Bicas‐Foz da Fonte section. In present forms, Doi et al. (2008) found out for the portunid crab *Charybdis bimaculata* studied in Tokyo Bay, Japan, that the proportion of males varied between 0.2–0.5 throughout the year and the overall sex ratio was greatly biased toward females. An overall higher mortality rate in male crabs compared to females can range from biological and ecological conditions.

#### 2.2.1 Mating Strategies and Behavior

In several species of portunid crabs, males tend to be more aggressive or have higher levels of activity due to their role in mating. This increased activity or aggression can lead to higher risk‐taking behaviors, such as fighting for territory, shelter, or mates, predation, or other stressors, which can increase mortality in males. Males may also be more vulnerable during certain parts of their life cycle, such as during mating seasons when they are more visible and exposed. For example, Yu et al. (2022) found in aquarium observations that there was cannibalism during the reproductive molting of female *P. trituberculatus* crabs, which could lead to the death or injury of some males. Shelters have the capability of appealing to this species during mating and can effectively reduce the incidence of intraspecific cannibalism during mating and improve the survival rate.

#### 2.2.2 Predation Pressure

Males may be more vulnerable to predation, especially during the mating season when they are more active and occupy certain regions where predators are abundant.

#### 2.2.3 Environmental Stress

Males might be more susceptible to environmental stresses such as changes in salinity and water temperature, due to their specific physiological or behavioral traits. Key stressors include thermal extremes impacting metabolism, acidification hindering growth, and habitat changes forcing range shifts, with physiological trade‐offs shaping their tolerance (Sousa et al. 2020). For example, males might spend more time in shallow waters during mating or foraging, which could expose them to fluctuating environmental conditions that might not affect females as much, as they often move to deeper waters for spawning (Silva et al. 2017).

#### 2.2.4 Parasites and Diseases

Male crabs might be more prone to certain parasites or diseases. In some species, males might have weaker immune responses or be more exposed to pathogens due to their behavior or ecology, leading to higher mortality rates. Disease outbreaks can sometimes have gendered effects, particularly if certain sexes are more likely to be in contact with infected individuals or environments. Kobayashi and Vazquez‐Archdale (2018) realized that the infection of the Rhizocephalan barnacle *Heterosaccus papillosus* on *Charybdis japonica* caused serious damage by making crabs lose their reproductive ability entirely in both sexes; especially, female host crabs live less than the males because they have to spend a large amount of energy on parasite reproduction. These impacts on the hosts are different from other previously reported rhizocephalan parasites; for example, in the case of *Sacculina granifera*, which infects *Portunus pelagicus* , parasites induce castration for all male hosts, but some female hosts show maturation of small gonads and ovipositioning.

#### 2.2.5 Reproductive Cycle

The timing of reproduction can also play a role. Male crabs often die shortly after mating, especially in species where they exert a lot of energy during the mating process or where they engage in dangerous behaviors to secure mates. In species where males die post‐mating, like *P. pelagicus* and *P. trituberculatus*, there is a higher mortality rate among males compared to females during certain times of the year (Xiao and Kumar 2004; Yu et al. 2022).

#### 2.2.6 Hormonal and Physiological Differences

Hormonal differences between males and females could make males more vulnerable to certain conditions, including stress, environmental changes, or injury (Herrera et al. 2024). For example, testosterone or other male‐specific hormones might contribute to more risky behavior that results in higher mortality (Soundarapandian 2013).

These factors, individually or in combination, can contribute to an overall higher mortality rate in male portunid crabs compared to females, especially during certain periods in their life cycle or under specific environmental conditions. In the case of Bicas‐Foz da Fonte section, the preservation of different beds with abundant nodules preserving nearly complete *A. monspeliensis* might suggest environmental stressors affecting dramatically the physiology of generations of mainly males, such as sudden changes in certain, but unknown ecological parameters. On the other hand, the occurrence of some autotomized crabs and only one clear evidence of injuries could indicate aggressive/defensive behavior during mating or eventual predation pressure, although the great majority of the crabs retrieved in nodules do not show evidence for predation. The death of these crabs, together with a significantly lower number of females, could have resulted from the mating process and happening for males immediately after mating, representing the beds with nodules the end of reproductive cycles for *A. monspeliensis* . Causes of generalized death related to hormonal imbalance, diseases, or parasite infestations seldom can be determined from the fossil record, but cannot be discarded either.

## 3 Conclusions

This study documents compelling fossil evidence of cheliped autotomy followed by regeneration in the Miocene swimming crab *Achelous monspeliensis*. Among an exceptional Burdigalian assemblage from the Bicas–Foz da Fonte section in Portugal, one specimen (CPM–BFF001D) displays marked dimensional asymmetry between the chelipeds, and CPM‐BFF009D individual presents a smaller and malformed carpus, consistent with a partially regenerated limb. The lack of deformity and the proportional reduction of all segment lengths in CPM‐BFF001D indicate a normal regenerative trajectory interrupted by the death of the individual prior to completing the molts required for full restoration. The results align with regenerative patterns observed in modern portunids and expand the fossil record of defensive behavior in decapods, previously limited to circumstantial examples.

By demonstrating that autotomy and early‐stage regeneration can be preserved in fossil crabs, this study provides a new behavioral and physiological insight into Miocene decapods, highlights the paleoecological significance of the Bicas–Foz da Fonte site assemblage, and underscores the value of exceptionally preserved crabs found in carbonate nodules for understanding the life histories of extinct portunids. The exceptional three‐dimensional, mostly complete preservation of crabs at this locality, combined with a few cases of retained exocuticle, suggests successive events of in situ high‐rate mortality among *A. monspeliensis*. This mortality more likely affecting male individuals was possibly caused by environmental stressors or highly‐energetic, highly aggressive mating behaviors representing the end of reproductive cycles, though the exact causes remain unknown.

## 4 Systematic Paleontology

Infraorder Brachyura Latreille

Subsection Heterotremata Guinot

Superfamily Portunoidea Rafinesque‐Schmaltz

Family Portunidae Rafinesque‐Schmaltz

Genus *Achelous* De Haan 1833

Type species: *Achelous spinimanus* Latreille 1819

*A. monspeliensis* (Milne Edwards 1860)

Figures 3 and 4

1884. *Achelous delgadoi*; Fontannes, p. 35, Pl. 7, fig. 1–8.

1954. *Neptunus granulatus*; Veiga Ferreira, p. 63, Pl. 1, figs. 1, 4, 7; Pl. 2, figs. 8–14; Pl. 3, figs. 18, 23; Pl. 4, figs. 31–32; Pl. 6, figs. 42, 44.

1959. *Neptunus granulatus*; Galopim de Carvalho, p. 80, Pl. 1; Pl. 2, figs. 1–11.

1962. *Neptunus granulatus*; Zbyszewski and Veiga Ferreira, p. 286, Pl. I, fig. 6.

1965. *Neptunus granulatus*; Veiga Ferreira, p. 150.

1979. *Portunus granulatus* ; Förster, p. 94.

1984. *Portunus monspeliensis*; Müller, p. 79, Pl. 62, figs. 1–2.

2016. *Portunus monspeliensis*; Gašparič et al., p. 58.

2022. *Portunus monspeliensis*; Neto de Carvalho and Marrecas, p. 12–13, fig. 1.

### 4.1 Description

The collection of 36 specimens examined represents adult individuals coming from different beds and of varying ages, with maximum carapace widths ranging from 42.5 to 76 mm (mean 56.6 ± 9.6 mm). The carapace in *A. monspeliensis* is quasi‐hexagonal, markedly wider than long (mean width/length ratio 1.68), with maximum width measured at the ninth anterolateral spine (Table 1). Moreover, the width/length ratio is variable (between 1.56 and 1.88) irrespective of the molting instar and the sex in the different populations. The carapace is slightly convex dorsally and ornamented with fine granulation.

The frontal margin projects beyond the frontorbital margin, situated slightly lower, and features a median notch flanked by two small forward‐pointing spines, followed by an inward‐directed orbital spine on each side. The frontorbital margin corresponds to 56% (mean = 31.7 ± 4.33 mm) of the maximum carapace width. The supraorbital margin is sinuous, with two closed fissures, one medial and one near the outer orbital tooth.

The anterolateral margin is long and gently convex, bearing nine spines: the first, positioned just outside the orbital region, is robust and anteriorly directed; it is followed by seven progressively smaller subtriangular spines oriented outward; the ninth spine is prominent and sharply pointed, reaching ~8 mm in length, laterally directed, and nearly perpendicular to the vertical axis. The posterolateral margin is straight, terminating in a concave depression that accommodates movement of the fifth pereiopod. The posterior margin is broad, slightly concave, and bordered, corresponding to approximately 39% of the frontorbital width.

Carapace regions are well defined: the protogastric region has semicircular lobes with a transverse ridge; mesogastric and metagastric regions are subtrapezoidal and separated by a weak transverse ridge; the mesogastric process is elongated, extending posteriorly beyond the frontal axial notch. The cardiac region is pentagonal, with a shallow longitudinal depression, straight anterior margins, and slightly concave posterior margins. The intestinal region is faintly defined and circular; the hepatic region is flat and triangular; the epibranchial region forms an arched crest extending from the ninth spine to the mesogastric region, separating the epi‐ and mesobranchial areas. The cervical groove and branchiocardiac grooves are clearly discernible.

On the ventral surface, the thoracic sternum is broad, oval, and widens posteriorly to the sixth sternite, with narrow and partially indistinct sutures. Sternites 1–2 are absent; sternites 3–4 are fused into a trapezoidal plate with an axial groove reaching the anterior margin of sternite 3 and a transverse ridge interrupted medially on sternite 4. Sternites 5–7 are transversely elongated, distally rounded, and laterally expanded; sternite 6 is the longest, sternite 7 is shorter, and sternite 8 is reduced and subtriangular. Sutures 4/5 to 7/8 are complete laterally.

It is not clear from the studied sample if *A. monspeliensis* shows sexual dimorphism related to size. The width range variation is broader in males than in females. In general, the genus *Achelous* shows sexual dimorphism, primarily in size, with males often larger than females, and in morphology, especially the chelipeds and abdomen, with males developing larger claws for competition and females having wider, egg‐carrying abdomens. However, *A. spinicarpus* can vary, sometimes showing no significant size difference, but distinct claw/abdomen features. Males often have more developed carpal spines on their claws for display, while females' abdominal width (pleon) grows differently, becoming broad and oval for egg‐carrying (Pardal‐Souza and Pinheiro 2013). In the male specimens of *A. monspeliensis* (Figure 4), the abdomen is subtriangular, with straight, converging margins. Somites 1 and 2 are narrow and broad, respectively; only somite 2 is visible ventrally, exhibiting a distal concave notch articulating with thoracic sternite 8. Somites 3–5 are fused into a broad subtrapezoidal plate with slightly concave lateral margins and transverse ridges. Somite 6 is trapezoidal, longer than somites 3–5, and the telson is subtriangular, as long as wide.

The female abdomen is considerably broader, reaching half the carapace width, with a subcircular outline. Somites 3–5 are rectangular with convex lateral margins; somite 6 widens posteriorly with a sinuous lateral margin, convex posteriorly and concave anteriorly; the telson is triangular and rounded. In the sample the male/female ratio is 2.6:1.

All specimens exhibit the typical homochely (Figure 4A), with differences between cheliped segments of 0–2 mm, except CPM‐BFF001D that shows pronounced asymmetry, with the left cheliped segments significantly smaller than the right one (Figure 4B,C). It is also to remark from the measurements the specimens CPM‐BFF010D, with the proximal segment of the right cheliped (merus) apparently smaller than the left one; however, in this case the left cheliped is more distended and rotated to a lower plane (Figure 4D). CPM/BFF009D also presents the left carpus smaller than the right one (see below and Figure 4H for discussion). In general, the merus is elongated, bearing two anteriorly directed spines and a third spine directed upward. The carpus is shorter and bears a large inward‐pointing spine. The palm is rectangular, with three longitudinal ridges on the outer surface and more pronounced granulation than the carapace. The propodus is triangular, elongated, and approximately equal in length to the palm. The occlusal margin of the chelae is heterodont, with a knob‐like molariform tooth on the right chela followed by a row of tuberculate teeth.

### 4.2 Remarks

Fontannes (1884) described for the first time the species *Achelous delgadoi*, which was later ascribed to *Portunus* (Schweitzer et al. 2010), from the Burdigalian Prazeres Beds cropping out in Lisbon (Pais et al. 2006). Meanwhile, Veiga Ferreira (1954, 1965) reported more specimens of this species as well as remains of *Neptunus granulatus* A. Milne‐Edwards, 1860 (=*Neptunus monspeliensis* A. Milne‐Edwards, 1860), both in Lisbon and in the Penedo‐Bicas‐Foz da Fonte coastal section. By assigning the Portuguese specimens to *Achelous monspeliensis* we follow the new combination of Hyžný and Dulai (2021) according with the suggestions of Spiridonov (2020, 158) to make the fossil taxa compatible with the recent rearrangement of extant Portunoidea classification based on morphological revision and molecular phylogenetic reconstructions (Mantelatto et al. 2007, 2018; Schubart and Reuschel 2009; Spiridonov et al. 2014; Evans 2018).

In the sites where it is known, the preservation of *A. monspeliensis* is commonly poor, usually limited to chelae or movable digits, or to carapaces that are poorly preserved, compressed by sedimentary compaction, or fractured (Gašparič and Ossò 2016; Díaz‐Medina et al. 2018). The occurrence of fully preserved specimens, consistently retaining both chelipeds and the proximal segments of most pereiopods, at the Bicas‐Foz da Fonte site, is exceptional. Such preservation is comparable only to occurrences in Middle Miocene marly limestones of Sardinia (Marangon and De Angeli 2009), which display complete carapaces and the preservation of the exocuticle with evidence of original ornamentation and pigmentation. In the specimens presented here, exocuticle preservation is generally absent, although at least three specimens from the study area do show such exceptional preservation.

The genera *Portunus* and *Achelous* include about 80 extant species altogether (Mantelatto et al. 2007; Karasawa et al. 2008; Schweitzer et al. 2010). *A. monspeliensis* is the most abundant crab species in Burdigalian formations (potentially extending into the Middle Miocene at Penedo) in Portugal, and it is typically the dominant decapod wherever it occurs (Gašparič and Ossò 2016), of which the Penedo and Bicas‐Foz da Fonte sections are not exceptions. The species had a broad paleogeographic distribution during the Miocene, inhabiting subtidal environments along the Atlantic, Mediterranean, and Paratethys margins. It has been documented in formations in Austria, France, Hungary, Italy, Malta, Portugal, Spain, Slovenia, Bosnia, Egypt, and the Sinai Peninsula (Gašparič and Ossò 2016; Gašparič et al. 2019).

Morphologically, *A. monspeliensis* resembles the extant *P. pelagicus* , currently found along the eastern Mediterranean margins, which suggests similar ecological preferences and behaviors between these species (Lai et al. 2010; Kunsook et al. 2014). Accordingly, *A. monspeliensis* would have been an euryhaline species, inhabiting coastal, estuarine, and lagoonal environments at depths of up to 30 m (Müller 1984; Gašparič and Ossò 2016). It was an active swimmer, using the fifth pair of pereiopods as oars, and likely remained buried in the substrate between feeding periods. Functionally, it acted as an opportunistic predator and scavenger, utilizing the right chela, which bears a knob‐like molariform tooth followed by tuberculate or conical teeth, to crush and dismember prey (Spiridonov et al. 2014).

## Author Contributions

**Carlos Neto de Carvalho:** conceptualization (lead), formal analysis (lead), funding acquisition (lead), investigation (lead), methodology (lead), project administration (lead), resources (equal), supervision (lead), validation (equal), visualization (equal), writing – original draft (lead), writing – review and editing (equal). **Pedro Marrecas:** writing – original draft (equal).

## Funding

This work was funded by FCT, I.P./MCTES (PT) through national funds (PIDDAC): LA/P/0068/2020 (https://doi.org/10.54499/LA/P/0068/2020), UID/50019/2025 ‐ (https://doi.org/10.54499/UID/50019/2025), and by the European Union ‐ NextGenerationEU under projects UID/PRR/50019/2025 (https://UID/doi.org/10.54499/PRR/50019/2025) and UID/PRR2/50019/2025 (https://doi.org/10.54499/PRR2/50019/2025). CNC also acknowledges the financial support provided by the Institute of Earth Sciences (ICT) through the multi‐annual funding contract with the Foundation for Science and Technology (FCT), under project UID/04683 with the DOI https://doi.org/10.54499/UID/04683/2025.

## Conflicts of Interest

The authors declare no conflicts of interest.

## Data Availability

All data generated or analyzed during this study are included in this published article. The collection of *A. monspeliensis* is made available for further studies by P.M. upon request by email.

## References

[ece373799-bib-0001] Allison, P. A. 1986. “Soft‐Bodied Animals in the Fossil Record: The Role of Decay in Fragmentation During Transport.” Geology 14: 979–981.

[ece373799-bib-0002] Ameer Hamsa, K. M. S. 1982. “Observationts [Sic!] on Moulting of Crab *Portunus pelagicus* Linnaeus Reared in the Laboratory.” Journal of the Marine Biological Association of India 24: 69–71.

[ece373799-bib-0003] Antunes, M. T. , J. Civis , J. A. González‐Delgado , P. Legoinha , A. Nascimento , and J. Pais . 1998. “Lower Miocene Stable Isotopes (δ18O, δ13C), Biostratigraphy and Environments in the Foz da Fonte and Penedo Sections (Setúbal Peninsula, Portugal).” Geogaceta 23: 7–10.

[ece373799-bib-0004] Antunes, M. T. , H. Elderfield , P. Legoinha , A. Nascimento , and J. Pais . 1999. “A Stratigraphic Framework for the Miocene From the Lower Tagus Basin (Lisbon, Setúbal Peninsula, Portugal). Depositional Sequences, Biostratigraphy and Isotopic Ages.” Revista de la Sociedad Geológica de España 12, no. 1: 3–15.

[ece373799-bib-0005] Antunes, M. T. , H. Elderfield , P. Legoinha , and J. Pais . 1995. “Datações isotópicas com Sr do Miocénico do flanco Sul da Serra da Arrábida.” Comunicações Do Instituto Geológico e Mineiro 81: 73–78.

[ece373799-bib-0006] Baucon, A. , M. Avanzini , C. Neto de Carvalho , Z. Belaústegui , N. B. Preto , and A. Reda . 2025. “The Earliest Evidence of True Crabs? Insights on the Evolution of Brachyura From an Exceptional Exposure of Carnian *Psilonichnus* (Upper Triassic, Braies, Dolomites, Italy).” Palaeogeography, Palaeoclimatology, Palaeoecology 667: 112820.

[ece373799-bib-0007] Behrensmeyer, A. K. , S. M. Kidwell , and R. A. Gastaldo . 2000. “Taphonomy and Paleobiology.” Paleobiology 26: 103–147.

[ece373799-bib-0008] Benton, M. J. 2010. “Studying Function and Behavior in the Fossil Record.” PLoS Biology 8, no. 3: e1000321. 10.1371/journal.pbio.1000321. 20209139 PMC2830450

[ece373799-bib-0009] Bliss, D. E. 1960. “Autotomy and Regeneration.” In The Physiology of Crustacea, edited by T. H. Waterman , vol. 1, 561–589. Academy Press.

[ece373799-bib-0010] Cachão, M. , and C. M. da Silva . 2000. “The Three Main Marine Depositional Cycles of the Neogene of Portugal.” Ciências da Terra (UNL) 14: 303–312.

[ece373799-bib-0011] Carmona, N. B. , L. A. Buatois , and M. G. Mángano . 2004. “The Trace Fossil Record of Burrowing Decapod Crustaceans: Evaluating Evolutionary Radiations and Behavioural Convergence.” Fossils and Strata 51: 141–153.

[ece373799-bib-0012] Charbonnier, S. , D. Pérès , and C. Letenneur . 2012. “Exceptionally Preserved Crustaceans From the Oxfordian of Eastern France (Terrain à Chailles Formation, Haute‐Saône).” Geodiversitas 34, no. 3: 531–568.

[ece373799-bib-0013] De Baets, K. , and D. T. J. Littlewood . 2015. “The Importance of Fossils in Understanding the Evolution of Parasites and Their Vectors.” Advances in Parasitology 90: 1–51. 26597064 10.1016/bs.apar.2015.07.001

[ece373799-bib-0014] De Haan, W. 1833–1850. “Crustacea.” In *Fauna Japonica sive Descriptio Animalium, Quae in Itinere per Japoniam, Jussu et Auspiciis Superiorum, qui Summum in India Batava Imperium Tenent, Suscepto, Annis 1823–1830 Collegit, Noitis, Observationibus et Adumbrationibus Illustravit*, edited by P. F. von Siebold, 243. Leiden, Lugduni‐Batavorum.

[ece373799-bib-0015] Díaz‐Medina, G. , À. Ossó , and M. Hyžný . 2018. “A Middle Miocene Decapod Faunule From Granada (Spain), with Remarks on Distribution Pattern of the Crab *Portunus monspeliensis* .” Neues Jahrbuch für Geologie und Paläontologie Abhandlungen 288: 129–141.

[ece373799-bib-0016] Doi, W. , M. Yokota , C. A. Strüssmann , and S. Watanabe . 2008. “Growth and Reproduction of the Portunid Crab *Charybdis bimaculata* (Decapoda: Brachyura) in Tokyo Bay.” Journal of Crustacean Biology 28, no. 4: 641–651.

[ece373799-bib-0017] Evans, N. 2018. “Molecular Phylogenetics of Swimming Crabs (Portunoidea Rafinesque, 1815) Supports a Revised Family‐Level Classification and Suggests a Single Derived Origin of Symbiotic Taxa.” PeerJ 6: e4260. 29379685 10.7717/peerj.4260 PMC5786103

[ece373799-bib-0018] Feldmann, R. M. 2003. “Interpreting Ecology and Physiology of Fossil Decapod Crustaceans.” Contributions to Zoology 72, no. 2–3: 111–117.

[ece373799-bib-0019] Figueiredo, S. , C. Neto de Carvalho , M. Cachão , and A. Fonseca . 2023. “A Marine Bird (Sulidae, Aves) From the Langhian (Middle Miocene) of Penedo Beach (Setúbal Peninsula—SW Portugal) and Its Paleoenvironmental Context.” Journal of Iberian Geology 49: 21–29.

[ece373799-bib-0020] Fontannes, F. 1884. “Note sur quelques gisements nouveaux des Terrains Miocènes du Portugal et description d'un Portunien du genre *Achelous*.” Paris.

[ece373799-bib-0021] Förster, R. 1969. “Epökie, entökie, parasitismus und regeneration bei fossilen dekapoden.” Bayerische Staatssammlung für Paläontologie Und Historische Geologie 9: 45–59.

[ece373799-bib-0022] Förster, R. 1979. “Decapod Crustaceans From the Middle Miocene (Badenian) Deposits of Southern Poland.” Acta Geologica Polonica 29: 89–106.

[ece373799-bib-0023] Fu, Y.‐Y. , L. Liu , C.‐R. Mu , et al. 2017. “On Limb Regeneration in Early Stage of *Portunus trituberculatus* in Histomorphology.” Oceanologia et Limnologia Sinica 48, no. 5: 1084–1091.

[ece373799-bib-0024] Galopim de Carvalho, A. M. 1959. “Malacostráceos das formações glauconiosas do Miocénico superior do litoral a norte do Cabo Espichel.” Boletim Do Museu e Laboratório Mineralógico e Geológico da Faculdade de Ciências da Universidade de Lisboa 8: 77–82.

[ece373799-bib-0025] Gašparič, R. , M. Hyžný , G. Jovanović , S. Ćorić , and S. Vrabac . 2019. “Middle Miocene Decapod Crustacean Assemblage From the Tuzla Basin (Tušanj, Bosnia and Herzegovina), with a Description of Two New Species and Comparison With Coeval Faunas From Slovenia.” Palaeontologia Electronica 22.1.9A: 1–21. 10.26879/894.

[ece373799-bib-0026] Gašparič, R. , and À. Ossò . 2016. “New Reports of Decapod *Portunus monspeliensis* A. Milne Edwards, 1860 From Miocene Beds of Eastern Slovenia With Notes on Palaeoecology and Palaeobiogeography.” Geologija 59, no. 1: 55–66.

[ece373799-bib-0027] Gibert, J. M. , F. Muñiz , Z. Belaústegui , and M. Hyžný . 2013. “Fossil and Modern Fiddler Crabs (*Uca tangeri*: Ocypodidae) and Their Burrows From SW Spain: Ichnologic and Biogeographic Implications.” Journal of Crustacean Biology 33, no. 4: 537–551.

[ece373799-bib-0028] He, J. , Y. Gao , W. Wang , et al. 2016. “Limb Autotomy Patterns in the Juvenile Swimming Crab (*Portunus trituberculatus*) on Earth Ponds.” Aquaculture 463: 189–192.

[ece373799-bib-0029] Herrera, I. , G. F. Carvalho‐Souza , and E. González‐Ortegón . 2024. “Physiological Responses of the Invasive Blue Crabs *Callinectes sapidus* to Salinity Variations: Implications for Adaptability and Invasive Success.” Comparative Biochemistry and Physiology Part A: Molecular & Integrative Physiology 297: 111709. 10.1016/j.cbpa.2024.111709 39053764

[ece373799-bib-0030] Hopkins, P. M. 2001. “Limb Regeneration in the Fiddler Crab *Uca pugilator* : Hormonal and Growth Factor Control.” American Zoologist 41: 389–398.

[ece373799-bib-0031] Hyžný, M. , and A. Dulai . 2021. Badenian Decapods of Hungary, 296. GeoLitera Publishing House, Institute of Geosciences, University of Szeged.

[ece373799-bib-0032] Hyžný, M. , C. S. Melo , R. S. Ramalho , et al. 2020. “Pliocene and Late Pleistocene (MIS 5e) Decapod Crustaceans From Santa Maria Island (Azores Archipelago: Central Atlantic): Systematics, Palaeoecology and Palaeobiogeography.” Journal of Quaternary Science 36, no. 1: 91–109.

[ece373799-bib-0033] Jagt, J. W. M. , B. W. M. van Bakel , D. Guinot , R. H. B. Fraaije , and P. Artal . 2016. “Fossil Brachyura.” In Treatise on Zoology—Anatomy, Taxonomy, Biology. The Crustacea, 9 (Part C), edited by P. Castro , P. Davie , D. Guinot , F. Schram , and C. von Vaupel Klein , 847–920. Brill.

[ece373799-bib-0034] Juanes, F. , and L. D. Smith . 1995. “The Ecological Consequences of Limb Damage and Loss in Decapod Crustaceans: A Review and Prospects.” Journal of Experimental Marine Biology and Ecology 193: 197–223.

[ece373799-bib-0035] Karasawa, H. , C. E. Schweitzer , and R. M. Feldmann . 2008. “Revision of Portunoidea Rafinesque, 1815 (Decapoda: Brachyura) With Emphasis on the Fossil Genera and Families.” Journal of Crustacean Biology 28, no. 1: 82–127.

[ece373799-bib-0036] Kidwell, S. M. , and K. W. Flessa . 1996. “The Quality of the Fossil Record: Populations, Species, and Communities.” Annual Review of Earth and Planetary Sciences 24: 433–464.

[ece373799-bib-0037] Kidwell, S. M. , F. T. Fürsich , and T. Aigner . 1986. “Conceptual Framework for the Analysis and Classifications of Fossil Concentrations.” PALAIOS 1: 228–238.

[ece373799-bib-0038] Klompmaker, A. A. , and G. A. Boxshall . 2015. “Fossil Crustaceans as Parasites and Dosts.” Advances in Parasitology 90: 233–289. 26597069 10.1016/bs.apar.2015.06.001

[ece373799-bib-0039] Klompmaker, A. A. , P. H. Kelley , D. Chattopadhyay , J. C. Clements , J. W. Huntley , and M. Kowalewski . 2019. “Predation in the Marine Fossil Record: Studies, Data, Recognition, Environmental Factors, and Behavior.” Earth‐Science Reviews 194: 472–520.

[ece373799-bib-0040] Klompmaker, A. A. , R. W. Portell , and M. G. Frick . 2017. “Comparative Experimental Taphonomy of Eight Marine Arthropods Indicates Distinct Differences in Preservation Potential.” Palaeontology 60: 773–794.

[ece373799-bib-0041] Kobayashi, S. , and M. Vazquez‐Archdale . 2018. “Growth and Reproductive Ecology of the Portunid Crab *Charybdis japonica* in an Open Seacoast and an Inland Bay in Fukuoka, Japan.” Journal of Sea Research 142: 52–65.

[ece373799-bib-0042] Koneru, M. , and T. Caro . 2025. “Defensive Behaviour in Intertidal Crabs.” Ethology 131, no. 4: e13544.

[ece373799-bib-0043] Kotchetoff, B. , Y. Kotchetoff , and O. Veiga Ferreira . 1975. “Contribution à la connaissance des gisements fossilifères miocènes au Nord du Cap d'Espichel.” Comunicações dos Serviços Geológicos de Portugal 59: 59–106.

[ece373799-bib-0044] Krause, R. A. , K. Parsons‐Hubbard , and S. E. Walker . 2011. “Experimental Taphonomy of a Decapod Crustacean: Long‐Term Data and Their Implications.” Palaeogeography, Palaeoclimatology, Palaeoecology 312: 350–362.

[ece373799-bib-0045] Kunsook, C. , N. Gajaseni , and N. Paphavasit . 2014. “The Feeding Ecology of the Blue Swimming Crab, *Portunus pelagicus* (Linnaeus, 1758), at Kung Krabaen Bay, Chanthaburi Province, Thailand.” Tropical Life Sciences Research 25, no. 1: 13–27. 25210585 PMC4156471

[ece373799-bib-0046] Lai, J. C. Y. , P. K. L. Ng , and P. J. F. Davie . 2010. “A Revision of the *Portunus pelagicus* (Linneus, 1758) Species Complex (Crustacea: Brachura: Portunidae), with the Recognition of Four Species.” Raffles Bulletin of Zoology 58, no. 2: 199–237.

[ece373799-bib-0047] Latreille, P. A. 1819. “Salicoques, Carides, Latr. In Nouveau Dictionnaire d'Histoire naturelle, appliquée aux arts, à l'Agriculture et à l'Économie rurale et domestique, à la Médecine, etc. Par une Société de Naturalistes et d'Agriculteurs.” Nouvelle Édition presq'entièrement refondue et considérablement augmentée; avec des figures tirées des trois Règnes de la Nature 30: 68–73.

[ece373799-bib-0048] Lee, S. Y. 2016. “Ecology of Brachyura.” In Treatise on Zoology—Anatomy, Taxonomy, Biology. The Crustacea, 9 (Part C), edited by P. Castro , P. Davie , D. Guinot , F. Schram , and C. von Vaupel Klein , 469–541. Brill.

[ece373799-bib-0049] Lindgren, J. , M. Heingård , C. Alwmark , et al. 2025. “Exceptionally Preserved Cretaceous Crabs Provide Novel Insights Into the Fossilization of Arthropod Compound Eyes.” Royal Society Open Science 12, no. 12: 250478. 10.1098/rsos.250478.

[ece373799-bib-0050] Liu, L. , Y. Fu , F. Zhu , et al. 2018. “Transcriptomic Analysis of *Portunus trituberculatus* Reveals a Critical Role for WNT4 and WNT Signalling in Limb Regeneration.” Gene 658: 113–122. 29524579 10.1016/j.gene.2018.03.015

[ece373799-bib-0051] Luque, J. , H. D. Bracken‐Grissom , J. Ortega‐Hernández , and J. M. Wolfe . 2024. “Fossil Calibrations for Molecular Analyses and Divergence Time Estimation for True Crabs (Decapoda: Brachyura).” Palaeontologia Electronica 27, no. 2: a38.

[ece373799-bib-0052] Luque, J. , R. M. Feldmann , O. Vernygora , et al. 2019. “Exceptional Preservation of Mid‐Cretaceous Marine Arthropods and the Evolution of Novel Forms via Heterochrony.” Science Advances 5: eaav3875. 31032408 10.1126/sciadv.aav3875 PMC6482010

[ece373799-bib-0053] Luque, J. , L. Xing , D. E. G. Briggs , et al. 2021. “Crab in Amber Reveals an Early Colonization of Nonmarine Environments During the Cretaceous.” Science Advances 7: eabj5689. 34669480 10.1126/sciadv.abj5689 PMC8528423

[ece373799-bib-0054] Mantelatto, F. L. , R. Robles , and D. L. Felder . 2007. “Molecular Phylogeny of the Western Atlantic Species of the Genus *Portunus* (Crustacea, Brachyura, Portunidae).” Zoological Journal of the Linnean Society 150: 211–220.

[ece373799-bib-0055] Mantelatto, F. L. , R. Robles , I. S. Wehrtmann , C. D. Schuhbart , and D. L. Felder . 2018. “New Insights Into the Molecular Phylogeny of the Swimming Crabs of the Genera *Portunus* Weber, 1795 and Achelous De Haan, 1833 (Brachyura: Portunidae) of the Americas.” Journal of Crustacean Biology 38: 190–197.

[ece373799-bib-0056] Manuppella, G. , M. T. Antunes , J. Pais , M. M. Ramalho , and J. Rey . 1999. Notícia explicativa da folha 38‐B Setúbal, Carta Geológica de Portugal na escala 1/50000, 143. Instituto Geológico e Mineiro.

[ece373799-bib-0057] Marangon, S. , and A. De Angeli . 2009. “Exceptionally Preserved Specimens of *Portunus monspeliensis* (A. Milne Edwards, 1860) (Brachyura, Portunidae) From the Miocene of Sardinia (Italy).” Atti Della Società Italiana di Scienze Naturali e del Museo Civico di Storia Naturale di Milano 150, no. I: 3–12.

[ece373799-bib-0058] Martini, E. 1971. “Standard Tertiary and Quaternary Calcareous Nannoplankton Zonation.” In Proceedings of the Second International Conference on Planktonic Microfossils, edited by A. Farinacci , vol. 2, 739–785. Tecnoscienza.

[ece373799-bib-0059] Maruzzo, D. , I. Bonato , C. Brena , G. Fusco , and A. Minelli . 2005. “Appendage Loss and Regeneration in Arthropods: A Comparative View.” In Crustacea and Arthropod Relationships, edited by S. Koenemann and R. A. Jenner , 215–245. Taylor & Francis.

[ece373799-bib-0060] Matos, S. A. , A. L. Castilho , L. A. C. Prado , et al. 2021. “Taphonomy and Ontogeny of the Brachyuran Crab *Exucarcinus gonzagai*, From the Lower Cretaceous (Aptian) Romualdo Formation, Araripe Basin, NE Brazil.” Journal of South American Earth Sciences 111: 103443.

[ece373799-bib-0061] Milne Edwards, A. 1860. “Histoire des Crustacés podophthalmaires fossiles et Monographie des Décapodes Macroures de la famille des Thalassinens.” Annales Des Sciences Naturelles, 4e série 14: 129–357.

[ece373799-bib-0062] Müller, P. 1984. “Decapod Crustacea of the Badenian.” Geologica Hungarica, Series Palaeontologica 42: 1–317.

[ece373799-bib-0063] Müller, P. , M. Krobicki , and G. Wehner . 2000. “Jurassic and Cretaceous Primitive Crabs of the Family Prosopidae (Decapoda: Brachyura) – Their Taxonomy, Ecology and Biogeography.” Annales. Societatis Geologorum Poloniae 70: 49–79.

[ece373799-bib-0064] Neto de Carvalho, C. 2016. “The Massive Death of Lobsters Smothered Within Their *Thalassinoides* Burrows: The Example of the Lower Barremian From Lusitanian Basin (Portugal).” Comunicações Geológicas 103, no. especial I: 143–152.

[ece373799-bib-0065] Neto de Carvalho, C. , and P. Marrecas . 2022. “Primeira evidência de autotomia com quelípode em regeneração no caranguejo *Portunus monspeliensis* do Miocénico Inferior de Portugal.” Boletim Do Centro Português de Geo‐História e Pré‐História 4, no. 1: 9–18.

[ece373799-bib-0066] Neto de Carvalho, C. , B. Pereira , A. Klompmaker , et al. 2016. “Running Crabs, Walking Crinoids, Grazing Gastropods: Behavioral Diversity and Evolutionary Implications of the Cabeço da Ladeira Lagerstätte (Middle Jurassic, Portugal).” Comunicações Geológicas 103, no. especial I: 39–54.

[ece373799-bib-0100] Neto de Carvalho, C. , N. P. Rodrigues , P. A. Viegas , A. Baucon , and V. F. Santos . 2010. “Patterns of occurrence and distribution of crustacean ichnofossils in the Lower Jurassic‐Upper Cretaceous of Atlantic occidental margin basins, Portugal.” Acta Geologica Polonica 60: 19–28.

[ece373799-bib-0067] Neto de Carvalho, C. , P. Viegas , and M. Cachão . 2007. “ *Thalassinoides* and Its Producer: Populations of *Mecochirus* Buried Within Their Burrow Systems, Boca do Chapim Formation (Lower Cretaceous), Portugal.” PALAIOS 22: 107–112.

[ece373799-bib-0068] Ng, P. K. L. , D. Guinot , and P. J. F. Davie . 2008. “Systema Brachyurorum: Part I. An Annotated Checklist of Extant Brachyuran Crabs of the World.” Raffles Bulletin of Zoology 17: 1–286.

[ece373799-bib-0069] Pais, J. , P. P. Cunha , D. Pereira , et al. 2012. The Paleogene and Neogene of Western Iberia (Portugal). A Cenozoic Record in the European Atlantic Domain, 158. Springer Briefs in Earth Sciences.

[ece373799-bib-0070] Pais, J. , C. Moniz , J. Cabral , et al. 2006. Carta Geológica de Portugal na escala de 1:50 0000, Notícia Explicativa da Folha 34‐D Lisboa, 74. Instituto Nacional de Engenharia, Tecnologia e Inovação.

[ece373799-bib-0071] Pardal‐Souza, A. L. , and M. A. A. Pinheiro . 2013. “Relative Growth and Reproduction in *Achelous spinicarpus* (Crustacea: Portunidae) on the South‐Eastern Continental Shelf of Brazil.” Journal of the Marine Biological Association of the United Kingdom 93, no. 3: 667–674.

[ece373799-bib-0072] Paterson, B. , D. Mann , B. Kelly , and M. Barchiesi . 2007. “Limb‐Loss in Pond‐Reared Blue Swimmer Crabs *Portunus pelagicus* (L.): Effect of Growth in an Indoor Shedding System.” Aquaculture Research 38, no. 14: 1569–1579.

[ece373799-bib-0073] Santos, A. , E. Mayoral , C. Marques da Silva , M. Cachão , and J. C. Kullberg . 2010. “ *Trypanites* Ichnofacies: Palaeoenvironmental and Tectonic Implications. A Case Study From the Miocene Disconformity at Foz da Fonte (Lower Tagus Basin, Portugal).” Palaeogeography, Palaeoclimatology, Palaeoecology 292, no. 1–2: 35–43.

[ece373799-bib-0074] Schubart, C. D. , and S. Reuschel . 2009. “A Proposal for a New Classification of Portunoidea and Cancroidea (Brachyura: Heterotremata) Based on Two Independent Molecular Phylogenies.” In Decapod Crustacean Phylogenetics, edited by J. W. Martin , K. A. Crandall , and D. L. Felder , vol. 18, 533–550. Crustacean Issues.

[ece373799-bib-0075] Schweitzer, C. E. , R. M. Feldmann , A. Garassino , H. Karasawa , and G. Schweigert . 2010. Systematic List of Fossil Decapod Crustacean Species. Vol. 10, 1–222. Crustaceana Monographs.

[ece373799-bib-0076] Silva, A. P. , F. Rocha , P. A. R. R. Legoinha , J. J. C. Pais , M. T. Antunes , and C. Gomes . 2000. “Miocene Sediments From Foz da Fonte and Penedo Sections (Lower Tagus Basin): Clay Minerals and Isotopic Data.” Ciências da Terra 14: 191–202.

[ece373799-bib-0077] Silva, T. E. , F. G. Taddei , G. Bertini , L. S. Andrade , G. M. Teixeira , and A. Fransozo . 2017. “Population Structure of the Swimming Crab *Achelous spinicarpus* (Crustacea, Portunoidea) in São Paulo Northern Coast, Brazil.” Neotropical Biology and Conservation 12, no. 3: 164–170.

[ece373799-bib-0078] Smith, L. D. 1995. “Effects of Limb Autotomy and Tethering on Juvenile Blue Crab Survival From Cannibalism.” Marine Ecology Progress Series 116: 65–74.

[ece373799-bib-0079] Soundarapandian, P. 2013. “Reproductive Biology of the Commercially Important Portunid Crab, *Portunus sanguinolentus* (Herbst).” Journal of Marine Science Research & Development 3, no. 2: 1–9.

[ece373799-bib-0080] Sousa, A. N. , V. P. Bernardes , C. H. Bernardo , et al. 2020. “Reproductive Biology of the Swimming Crab *Achelous spinimanus* (Decapoda, Portunoidea): A Potential Fishing Resource.” Iheringia, Série Zoologia 110: e2020010.

[ece373799-bib-0081] Spiridonov, V. A. 2020. “An Update of Phylogenetic Reconstructions, Classification and Morphological Characters of Extant Portunoidea Rafinesque, 1815 (Decapoda, Brachyura, Heterotremata), with a Discussion of Their Relevance to Fossil Material.” Geologija 63, no. 1: 133–166.

[ece373799-bib-0082] Spiridonov, V. A. , T. V. Neretina , and D. Schepetov . 2014. “Morphological Characterization and Molecular Phylogeny of Portunoidea Rafinesque, 1815 (Crustacea Brachyura): Implications for Understanding Evolution of Swimming Capacity and Revision of the Family Level Classification.” Zoologischer Anzeiger 253: 404–429.

[ece373799-bib-0083] Triay‐Portella, R. , A. Escribano , J. Zarcero , F. Tuya , and J. G. Pajuelo . 2018. “Limb Autotomy in *Portunus hastatus* Population: Temporal, Sexual and Ontogenetic Variation.” In Abstracts Volume VI International Symposium on Marine Sciences, June 2018, edited by B. Rubio , A. M. Bernabeu , D. Rey , et al., 132. University of Vigo.

[ece373799-bib-0084] Tsang, L. M. , C. D. Schubart , S. T. Ahyong , et al. 2014. “Evolutionary History of True Crabs (Crustacea: Decapoda: Brachyura) and the Origin of Freshwater Crabs.” Molecular Biology and Evolution 31, no. 5: 1173–1187. 24520090 10.1093/molbev/msu068

[ece373799-bib-0085] Turner, J. S. 2004. “Extended Phenotypes and Extended Organisms.” Biology and Philosophy 19: 327–352.

[ece373799-bib-0086] Turner, L. M. 2016. “Physiological Adaptations to the Environment in Brachyura.” In Treatise on Zoology—Anatomy, Taxonomy, Biology. The Crustacea, 9 (Part C), edited by P. Castro , P. Davie , D. Guinot , F. Schram , and C. von Vaupel Klein , 165–183. Brill.

[ece373799-bib-0087] Veiga Ferreira, O. 1954. “Malacostráceos do Miocénico marinho de Portugal.” Comunicações dos Serviços Geológicos de Portugal 35: 57–78.

[ece373799-bib-0088] Veiga Ferreira, O. 1958. “Descoberta de ‘Calappa heberti’ no Tortoniano do Penedo (Cabo Espichel).” Comunicações dos Serviços Geológicos de Portugal 42: 203–207.

[ece373799-bib-0089] Veiga Ferreira, O. 1965. “Nova contribuição para o conhecimento dos Malacostráceos do Miocénico marinho de Portugal.” Comunicações dos Serviços Geológicos de Portugal 48: 141–155.

[ece373799-bib-0090] Vernet, G. , and M. Charmantier‐Daures . 1994. “Mue, autotomie et régénération.” In Traité de Zoologie, Vol. 7(1), edited by P. P. Grassé , 153–194. Masson.

[ece373799-bib-0091] Wallaard, J. , R. Fraaije , B. V. Bakel , and J. W. R. Jagt . 2020. “ *Calappilia erwinhartei*, a New Calappid Crab (Crustacea, Decapoda) From the Lower Miocene of Portugal.” Neues Jahrbuch für Geologie Und Paläontologie ‐ Abhandlungen 238, no. 2: 147–153.

[ece373799-bib-0092] Wehner, G. 1988. “Über die Prosoponiden (Crustacea, Decapoda) des Jura.” Dissertation zur Erlangung des Doktorgrades der Fakultät für Geowissenschaften der Ludwig‐Maximilians‐Universität zu München, 154.

[ece373799-bib-0093] Wolfe, J. M. , L. Ballou , J. Luque , et al. 2024. “Convergent Adaptation of True Crabs (Decapoda: Brachyura) to a Gradient of Terrestrial Environments.” Systematic Biology 73, no. 2: 247–262. 37941464 10.1093/sysbio/syad066 PMC11282366

[ece373799-bib-0094] Wolfe, J. M. , J. Luque , and H. D. Bracken‐Grissom . 2021. “How to Become a Crab: Phenotypic Constraints on a Recurring Body Plan.” BioEssays 43, no. 5: 2100020. 10.1002/bies.202100020 33751651

[ece373799-bib-0095] Wood, F. D. , and H. E. Wood . 1932. “Autotomy in decapod Crustacea.” Journal of Experimental Zoology 62: 1–55.

[ece373799-bib-0096] Xiao, Y. , and M. S. Kumar . 2004. “Sex Ratio, and Probability of Sexual Maturity of Females at Size, of the Blue Swimmer Crab, *Portunus pelagicus* Linneaus, Off Southern Australia.” Fisheries Research 68, no. 1–3: 271–282.

[ece373799-bib-0097] Yu, H. , L. Wan , Y. Peng , et al. 2022. “Observations of the Mating Behavior of *Portunus trituberculatus* and the Role of Shelters in Its Mating Process.” Aquaculture Reports 22: 100926.

[ece373799-bib-0098] Zbyszewski, G. 1967. “Contributions a l'etude du Miocene de la serra da Arrabida.” Comunicações dos Serviços Geológicos de Portugal 51: 37–148.

[ece373799-bib-0099] Zbyszewski, G. , and O. Veiga Ferreira . 1962. “La faune miocene de l'ile de Santa Maria (Açores).” Comunicações dos Serviços Geológicos de Portugal 46: 247–289.

